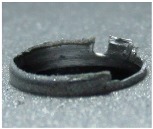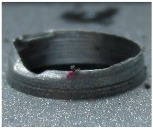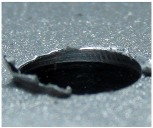# Investigation on the Effect of Drill Geometry and Pilot Holes on Thrust Force and Burr Height When Drilling an Aluminium/PE Sandwich Material

**DOI:** 10.3390/ma9090774

**Published:** 2016-09-13

**Authors:** Bruna Aparecida Rezende, Michele L. Silveira, Luciano M. G. Vieira, Alexandre M. Abrão, Paulo Eustáquio de Faria, Juan C. Campos Rubio

**Affiliations:** 1 Department of Production Engineering, Universidade Federal de Minas Gerais, Avenida Antônio Carlos, 6627, Pampulha, Belo Horizonte MG, CEP 31270-901, Brazil; brunarezende@ufmg.br (B.A.R.); michelesilveira1991@gmail.com (M.L.S.); lucianomgv@ufmg.br (L.M.G.V.); paulofaria@ufmg.br (P.E.d.F.); 2 Department of Mechanical Engineering, Universidade Federal de Minas Gerais, Avenida Antônio Carlos, 6627, Pampulha, Belo Horizonte MG, CEP 31270-901, Brazil; abrao@demec.ufmg.br

**Keywords:** drilling, sandwich composite, pilot hole, thrust force, burr height, analysis of variance

## Abstract

Composite materials are widely employed in the naval, aerospace and transportation industries owing to the combination of being lightweight and having a high modulus of elasticity, strength and stiffness. Drilling is an operation generally used in composite materials to assemble the final product. Damages such as the burr at the drill entrance and exit, geometric deviations and delamination are typically found in composites subjected to drilling. Drills with special geometries and pilot holes are alternatives used to improve hole quality as well as to increase tool life. The present study is focused on the drilling of a sandwich composite material (two external aluminum plates bound to a polyethylene core). In order to minimize thrust force and burr height, the influence of drill geometry, the pilot hole and the cutting parameters was assessed. Thrust force and burr height values were collected and used to perform an analysis of variance. The results indicated that the tool and the cutting speed were the parameters with more weight on the thrust force and for burr height they were the tool and the interaction between tool and feed. The results indicated that drilling with a pilot hole of Ø4 mm exhibited the best performance with regard to thrust force but facilitated plastic deformation, thus leading to the elevation of burr height, while the lowest burr height was obtained using the Brad and Spur drill geometry.

## 1. Introduction

Sandwich panels are structural composites with two outer sheets, or faces, which are separated by and adhesively bonded to a thicker core [1]. The use of sandwich materials is present in different industries such as civil, naval, aerospace and transportation. Advantages such as the ability to provide high bending stiffness, buckling and fatigue strength, and a lightweight structure are characteristics of sandwich panels and the most relevant property of sandwich composites in comparison with other laminate composites is their high strength-to-weight ratio [2].

Cutting operations such as drilling are frequently applied to sandwich materials in order to allow joining using bolts, rivets and screws. Typical drilling-related damage is: delamination, presence of burrs, circularity error and geometric damage, which can impair the performance of the finished component.

Delamination due to thrust force is one of most complex defects induced by drilling [3]. This problem has been defined as an inter-ply failure phenomenon [4]. According Khoran et al. [2], the feed rate is the factor that has the greatest influence on the delamination in a sandwich material. In the drilling of a sandwich panel there are at least two damaged regions in the top (push out) and bottom (peel up) in the delamination form [2,5]. In the study conducted by Tsao and Hocheng [6], the influence of drill geometry on delamination was investigated and the results showed that twist drills caused more severe damage in comparison with candlestick drills, core drills, saw drills and step drills.

Another difficulty found when drilling is the presence of burrs, especially when machining ductile materials. Burrs may occur at the entrance and/or at the exit of the hole as a consequence of uncut material which is plastically deformed [7].

Circularity deviations are often studied in order to assess hole quality. Circularity is a condition of a surface where all points of the surface intersected by any plane perpendicular to an axis are equidistant from that axis and circularity tolerance specifies a tolerance zone bounded by two concentric circles within which each circular element of the surface must lie [8]. Zitoune et al. [9] investigated the effect of drill diameter, spindle speed and feed rate on thrust force, torque, surface finish, circularity and hole diameter in the drilling of carbon fiber–reinforced plastic/aluminum stacks using cemented carbide drills (ISO grade K20). The results showed that the circularity error increased with the feed rate. In contrast, the spindle speed did not significantly affect the hole quality.

The machining process of thermoset polymers, such as polycarbonates, polymethylmethacrylate (acrylic glass), polyetheretherketone, and other semi-crystalline thermoplastics, must reach a specific temperature level (glass transition temperature) this high heat transfer can significantly affect the integrity of the material. Based on these observations, Lizardo et al. [10] reported that temperature plays a vital role in the machining of plastic material as it directly influences the rate of tool wear and the final surface finish of a workpiece material. In the last decade, the problems associated with the precision and efficiency in cutting composites have become an important issue in the manufacturing industry. Investigations on the machining mechanisms and production cost, such as those related to tool wear, selection of machining parameters and control of surface finish, have been carried out. In this way, much research has been reported to investigate the machinability of a wide variety of reinforced polymeric materials. However, few papers are available to discuss the machining parameters of engineering polymers and their effect on the surface finish [11].

In their work, Gutiérrez et al. [12] studied the influence of feed rate, cutting speed and tool geometry on thrust force, specific cutting pressure and hole dimensional deviations for drilling unreinforced polyamide (PA6) and on polyamide reinforced with 30 wt % glass fiber (PA66-GF30) using tungsten carbide drills (ISO grade K20) with different tip angles. The results clearly indicate the superior machinability of the reinforced material compared with the unreinforced polyamide due to the better physical and mechanical properties.

**Figure 1:**
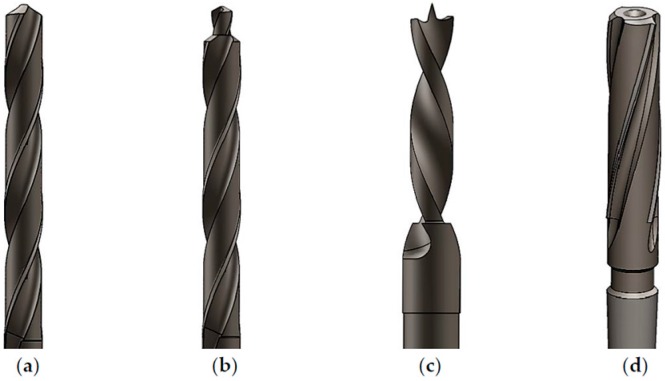
Selected drill geometries: (**a**) twist; (**b**) step; (**c**) Brad and Spur and (**d**) core drill.

In order to minimize the damage related to drilling, a range of cutting parameters and drill geometries together with a pilot hole is used. Figure 1a–d show typical drill geometries employed for the machining of composites: twist drill, step drill, Brad and Spur and core drill, respectively. One must bear in mind that damage in composites can be reduced when the machining forces are converged to the center of the drill, as in the case of the candlestick geometry.

Zitoune et al. [13] studied the drilling of a carbon fiber–reinforced polymeric composite using drills with different geometries in order to evaluate cutting forces and hole quality. It was found that the double cone drill geometry promoted lower thrust force values in comparison with a standard twist drill.

The effect of a pilot hole on thrust force when drilling carbon fiber–reinforced plastic using a saw drill was investigated by Tsao [14]. The pilot hole was used to eliminate the thrust caused by the chisel edge of the twist drill. The author concluded that a small ratio of the pilot hole to the drill diameter promotes early initiation of the bending mechanism and results in a large thrust force.

In this work, the drilling of a sandwich composite is studied and the thrust force and burr height have been measured. Distinct drill geometries and pilot holes were utilized. The influence of the tool geometry, pilot hole and drilling parameters was assessed by means of the analysis of variance.

## 2. Materials and Methods

The materials and experimental procedure used in this research work are depicted below.

### 2.1. Material

**Figure 2:**
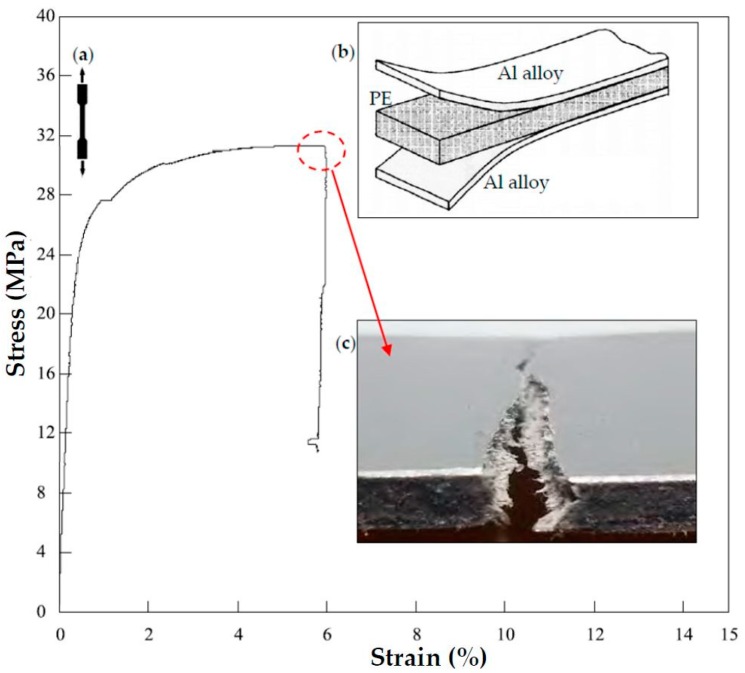
(**a**) Typical tensile curve of the sandwich composite; (**b**) Schematic diagram of the sandwich composite and (**c**) detail of a fractured specimen.

The investigated work material is a sandwich composite is made of a polyethylene (PE) core with density of 0.92 g/cm³ bound to two Al Mg 1 H2 (EN AW-ALMG-5005) aluminum alloy external plates. Figure 2 shows the typical behavior of the sandwich composite under tension with a detail of a fractured specimen and Table 1 summarizes the obtained mechanical properties (modulus of elasticity, tensile strength and elongation). The dimensions of the specimens are 100 mm × 120 mm × 4 mm.

### 2.2. Cutting Tools

**Figure 3:**
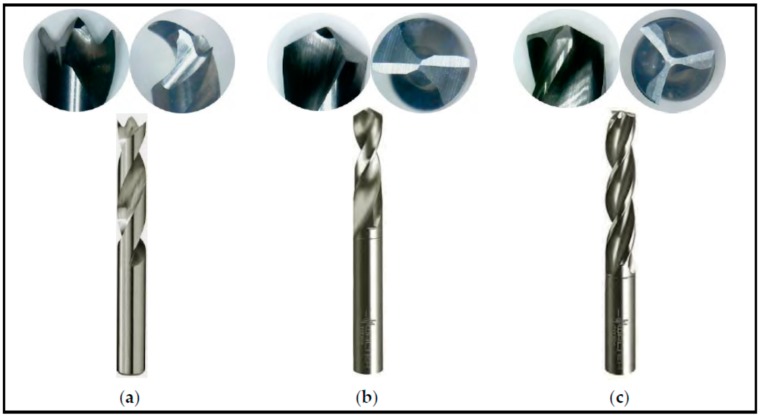
Drills used in the experiments: (**a**) Brad and Spur; (**b**) twist drill with two edges and (**c**) twist drill with three edges.

Tungsten carbide drills (length of 62 mm and diameter of 5 mm) with the following geometries and grades were tested as cutting tools: Brad and Spur (ISO grade K10), twist drill with two cutting edges and twist drill with three cutting edges (both ISO grade K30). When applicable, pilot holes with Ø2, Ø3 and Ø4 mm were drilled prior to tests using the twist drill with two edges. Figure 3 shows the geometries of the drills used in the experimental work.

### 2.3. Experimental Set-Up

**Figure 4:**
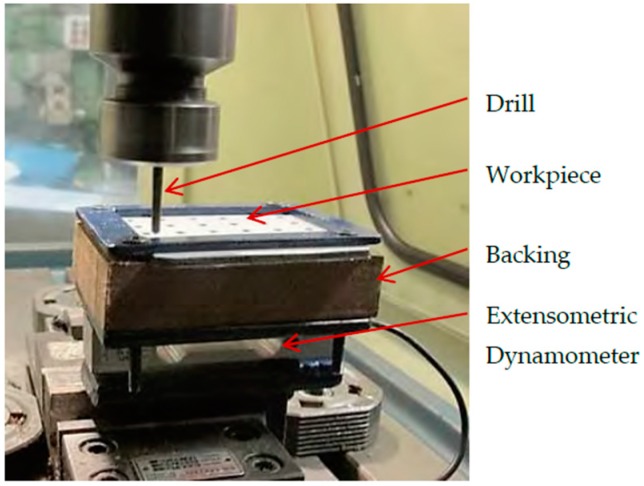
Experimental set-up for the drilling tests.

Drilling experiments were conducted on a machining center with 7.5 kW spindle power and maximum spindle speed of 7500 rpm. Thrust force was measured with an extensometric dynamometer. Burr height was assessed with a GSZ 2T toolmakers microscope (Askania, Rathenow, Germany). Table 2 presents the factors and levels employed in the experimental work and Figure 4 shows the experimental set-up. A full factorial experimental design was employed and each drilling condition was replicated once, thus resulting in 144 tests.

## 3. Results and Discussion

The Table 3 shows the results for the thrust force and burr height. The analysis of variance (ANOVA) was carried out, aimed at verifying whether the main factors and their interactions are statistically significant within a 95% confidence interval. *P*-values lower than or equal to 0.05 indicate that the main factor or the interaction significantly affects the studied response. These values are underlined in Table 4 and bold figures indicate significant factors of superior order which will be evaluated. Moreover, the correlation coefficient (*R*^2^ adj) for the model is presented. As far and the thrust force results are concerned, it can be noted that this parameter is statistically affected by all factors and their interactions, except for the interaction between tool, cutting speed and feed rate. In the case of burr height, the cutting speed is the only factor which does not possess a significant influence.

### 3.1. Thrust Force

**Figure 5:**
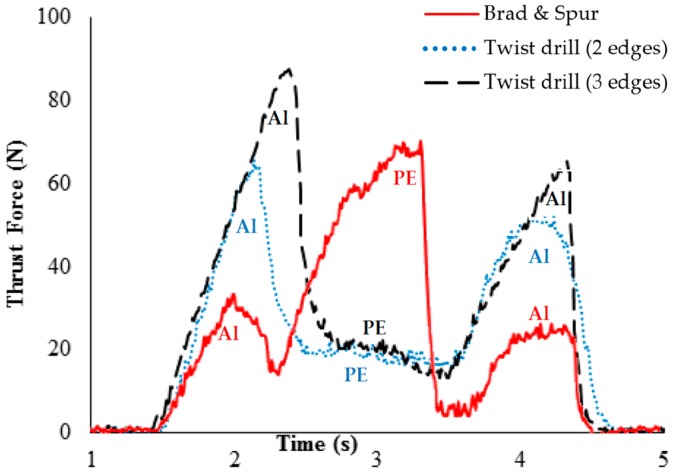
Profile of thrust force versus time for special drill bits for cutting speed 24 m/min and feed 0.05 mm/rev.

Figure 5 shows the behavior of the thrust force when drilling the composite sandwich using the twist drills (with two and three cutting edges) and the Brad and Spur drill. Despite the difference in values, for both twist drills the thrust force initially increases to reach its maximum as the first aluminum plate is cut, followed by a sudden decrease when the polymeric material is drilled due to its lower strength. The thrust force increases again when the second aluminum plate is drilled, however, with a considerably lower maximum due to the absence of backing. The thrust force for the twist drill with three edges was higher because its point angle is 150° while the point angle of the twist drill with two edges is 118°. A distinct trend is observed for the Brad and Spur drill: initially, while only the central cutting edge is in contact with the work material, the thrust force is the result of plastic deformation rather than shearing due to the null cutting speed at the center of the drill. The thrust force increases further to reach its maximum as the cutting edge penetrates the first aluminum plate, followed by a drastic decrease while the polyethylene layer is cut. A third peak of lowest intensity is observed when the second aluminum plate is drilled.

**Figure 6:**
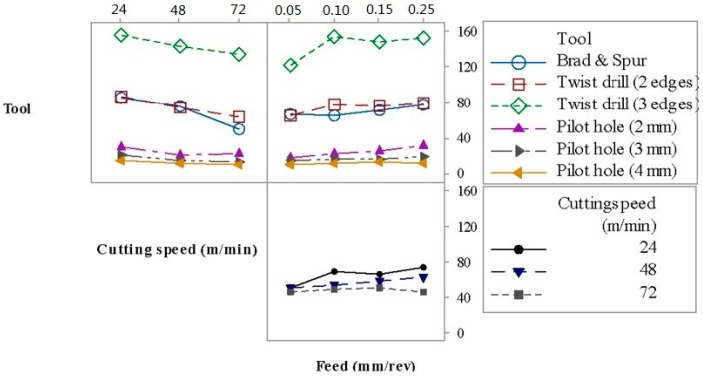
Interaction plot for thrust force.

Figure 6 shows the interaction plot for the thrust force. The principal factors, as well as the second-order interactions, significantly affect the thrust force. The tool and the cutting speed were the parameters with more weight on the thrust force. The increase in the cutting speed caused a decrease in the thrust force due to the elevation of temperature, which softens the matrix, thus reducing its strength, as reported by [15]. In general, an increase in the feed rate leads to a higher thrust force associated with the elevation of the shear area, similar to the results reported by [3]. Not surprisingly, drilling after generating a pilot hole required lower thrust force values and the higher the pilot hole diameter, the lower the force.

### 3.2. Burr Height

**Figure 7:**
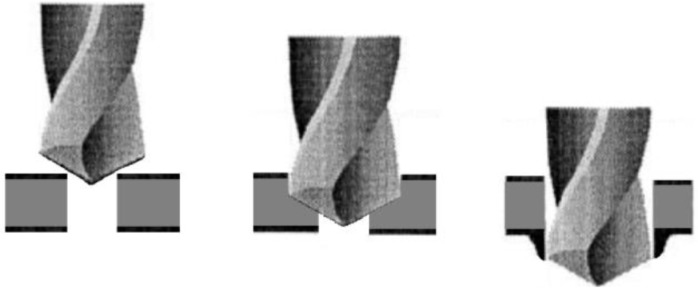
Burr formation when drilling using a pilot hole.

Burr occurs as a consequence of plastic deformation that takes place when the thickness of the material to be removed cannot withstand the acting forces. The costs and time associated with deburring are appreciable; therefore, it is necessary to optimize the cutting parameters, as well as the choice of the most suitable cutting tool, in order to avoid or minimize burr formation. Figure 7 schematically shows burr formation when drilling a sandwich composite with a pilot hole. The second aluminum plate cannot bear the thrust force and deforms plastically to generate the exit burr.

**Figure 8:**
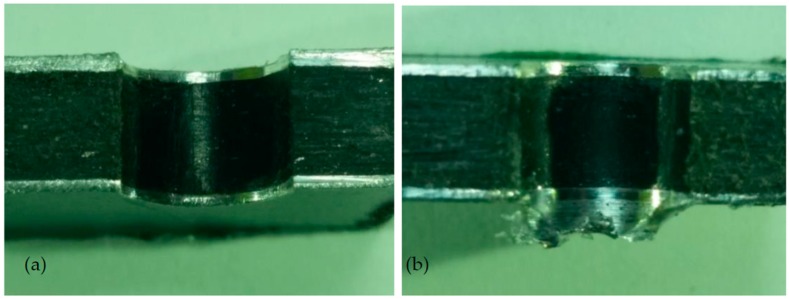
The cross-section of the sandwich material drilled, (**a**) without a burr and (**b**) with a burr.

Figure 8 shows the cross-section of the sandwich material, (a) without a burr and (b) with a burr in the exit, obtained due to lesser and greater feed, respectively.

Table 5 indicates that the tool geometry and feed rate are the significant factors affecting the burr height. Moreover, all the second- and third-order interactions are significant. The tool and the interaction between tool and feed were the parameters with more weight on the burr height. The interaction plot for the influence of the tool geometry, cutting speed and feed rate on the burr height is given in Figure 8, where it can be noticed that the presence of a pilot hole caused the burr height to increase. The Brad and Spur drill geometry promoted lower burr heights, followed by the twist drill with two cutting edges and by the twist drill with three cutting edges. Similar results were reported by Rubio et al. [16], who observed that Brad and Spur drills caused less damage (delamination) than twist drills.

**Figure 9:**
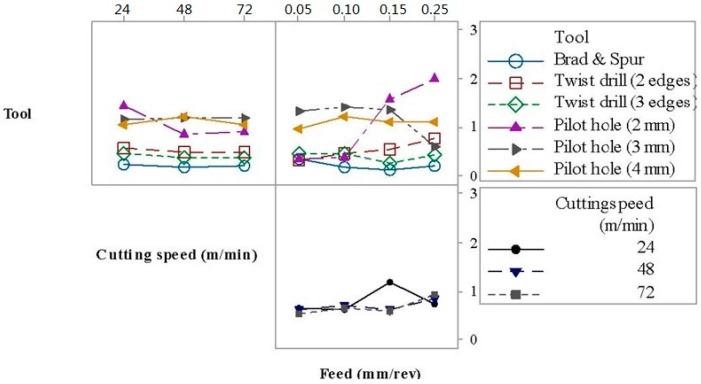
Interaction plot for burr height.

Figure 9 shows the interaction plot for burr height, where it can be inferred that the feed rate is the most influential factor because it does not lead to a linear trend, while the influence of the cutting speed is negligible (see Table 5). Furthermore, the deleterious influence of a pilot hole on burr height can be noted.

Table 5 shows the burrs formed when drilling the sandwich composite at a cutting speed of 48 m/min with various drill geometries and feed rates. The best results were obtained for the Brad and Spur geometry, irrespective of the tested feed rate. In the case of the twist drills, caps are present at the lowest feed rate and increasing the number of cutting edges leads to the formation of shorter burrs. Finally, drilling with a pilot hole results in higher burrs, as indicated in Figure 8.

## 4. Conclusions

After conducting drilling tests in a sandwich composite using three cutting speeds (24, 48 and 72 m/min), four feed rates (0.05, 0.10, 0.15 and 0.25 mm/rev) and three drill geometries (Brad and Spur and twist drills with two and three edges), with and without pilot holes (Ø2, Ø3 and Ø4 mm), the following conclusions can be drawn:

- All investigated factors and second-order interactions significantly affected thrust force. The thrust force decreased with the elevation of the cutting speed (due to work material softening) and increased with the feed rate (higher shear area). The Brad and Spur drill geometry required a lower thrust force compared with the twist drills with two and three cutting edges and the presence of a pilot hole contributed to the reduction of the thrust force.
- Drill geometry and feed rate, as well as all the second- and third-order interactions, were the factors that significantly affected the burr height. The Brad and Spur geometry was responsible for shorter burrs and the presence of a pilot hole facilitated plastic deformation, thus leading to the elevation of the burr height.

## Figures and Tables

**Table 1:** Mechanical properties of the sandwich composite.

Modulus of Elasticity (GPa)	Tensile Strength (MPa)	Tensile Strength (MPa)
6.51 ± 1.28	30.31 ± 1.78	4.5 ± 1.4

**Table 2:** Parameters and their levels.

Parameters	Levels					
	1	2	3	4	5	6
Drill geometry	Brad & Spur	Twist drill (2 edges)	Twist drill (3 edges)	Pilot hole (2 mm)	Pilot hole (3 mm)	Pilot hole (4 mm)
Cutting speed (m/min)	24	48	72	-	-	-
Feed (mm/rev)	0.05	0.10	0.15	0.25	-	-

**Table 3:** Results for thrust force and burr height.

Test	Tool	Cutting Speed (m/min)	Feed (mm/rev)	Thrust Force (N)		Burr Height (mm)	
1	1	1	1	66.76	70.53	0.62	0.23
2	1	1	2	79.99	84.85	0.27	0.13
3	1	1	3	104.11	77.87	0.18	0.09
4	1	1	4	94.95	98.43	0.15	0.10
5	1	2	1	75.45	75.27	0.31	0.16
6	1	2	2	69.25	59.96	0.15	0.08
7	1	2	3	94.66	48.54	0.08	0.11
8	1	2	4	77.44	98.87	0.25	0.16
9	1	3	1	55.66	55.28	0.28	0.34
10	1	3	2	50.03	47.70	0.09	0.17
11	1	3	3	42.30	55.21	0.10	0.06
12	1	3	4	46.84	43.23	0.16	0.37
13	2	1	1	65.77	75.52	0.14	0.65
14	2	1	2	88.47	89.03	0.45	0.56
15	2	1	3	85.28	86.98	0.47	0.77
16	2	1	4	96.68	96.04	0.69	0.72
17	2	2	1	65.02	66.62	0.22	0.24
18	2	2	2	74.20	76.42	0.34	0.69
19	2	2	3	77.62	77.04	0.59	0.46
20	2	2	4	73.27	78.97	0.62	0.69
21	2	3	1	57.86	60.51	0.34	0.21
22	2	3	2	65.20	68.02	0.31	0.25
23	2	3	3	65.12	63.23	0.40	0.49
24	2	3	4	63.43	63.08	0.79	0.97
25	3	1	1	87.58	148.10	0.33	0.36
26	3	1	2	184.21	185.51	0.23	0.36
27	3	1	3	127.62	165.18	0.45	0.15
28	3	1	4	176.49	170.67	0.13	1.57
29	3	2	1	98.65	144.13	0.76	0.36
30	3	2	2	121.70	159.45	0.31	0.61
31	3	2	3	144.45	164.17	0.18	0.30
32	3	2	4	147.02	164.05	0.22	0.13
33	3	3	1	118.66	136.72	0.19	0.57
34	3	3	2	153.27	124.28	0.31	0.91
35	3	3	3	142.99	141.65	0.19	0.15
36	3	3	4	123.76	129.25	0.31	0.15
37	4	1	1	17.95	20.21	0.61	0.47
38	4	1	2	24.11	27.28	0.22	0.28
39	4	1	3	37.69	31.09	3.50	3.52
40	4	1	4	44.81	33.59	1.46	1.44
41	4	2	1	17.31	15.91	0.19	0.28
42	4	2	2	18.36	17.89	0.38	0.37
43	4	2	3	18.15	18.93	0.61	0.49
44	4	2	4	25.48	29.54	2.20	2.23
45	4	3	1	16.42	16.23	0.20	0.20
46	4	3	2	20.14	21.53	0.44	0.50
47	4	3	3	22.44	22.35	0.64	0.62
48	4	3	4	26.30	23.53	2.54	2.10
49	5	1	1	14.54	15.78	1.66	1.23
50	5	1	2	16.70	19.18	0.87	1.60
51	5	1	3	19.88	20.46	1.48	1.42
52	5	1	4	26.53	24.00	0.70	0.17
53	5	2	1	12.16	12.71	2.19	0.54
54	5	2	2	13.53	12.70	1.63	1.43
55	5	2	3	14.35	15.38	1.39	1.45
56	5	2	4	16.18	15.90	0.20	0.63
57	5	3	1	11.72	12.36	0.82	1.43
58	5	3	2	12.24	12.74	1.48	1.38
59	5	3	3	12.80	11.98	1.16	1.22
60	5	3	4	10.60	12.04	1.09	0.71
61	6	1	1	10.73	10.73	0.94	0.81
62	6	1	2	11.50	15.30	1.50	0.98
63	6	1	3	13.45	17.59	1.30	1.05
64	6	1	4	12.48	16.21	0.61	1.04
65	6	2	1	8.98	7.94	1.24	0.93
66	6	2	2	10.32	10.80	1.17	1.58
67	6	2	3	9.50	11.35	0.70	1.17
68	6	2	4	9.93	11.35	0.78	2.16
69	6	3	1	7.37	8.72	0.89	0.95
70	6	3	2	9.33	9.32	1.07	0.98
71	6	3	3	8.24	14.92	1.16	1.12
72	6	3	4	7.28	7.97	0.85	1.14

**Table 4:** Analysis of variance (ANOVA) for thrust force and burr height.

Parameters	Thrust Force (N)			Burr Height (mm)		
	SS	F	*p*-Value	SS	F	*p*-Value
Tool	312.684	618.25	0.000	21.2177	52.77	0.000
Cutting speed	6617	32.71	0.000	0.4012	2.49	0.090
Feed	2879	9.49	0.000	1.3427	5.57	0.002
Tool * Cutting speed	3306	3.27	0.002	1.6056	2.00	0.046
Tool * Feed	2877	1.90	0.038	15.4322	12.79	0.000
Cutting speed * Feed	2059	3.39	0.005	2.7209	5.64	0.000
Tool * Cutting speed * Feed	2855	0.94	0.561	9.9944	4.14	0.000
*R*^2^ adj	95.75%			80.35%		

*: the interaction between the parameters.

**Table 5:** Burrs generated after drilling at a cutting speed of 48 m/min.

Tool	*f* *=* 0.05 mm/rev	*f* *=* 0.15 mm/rev	*f* *=* 0.25 mm/rev
Brad & Spur	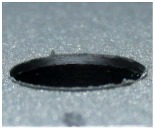	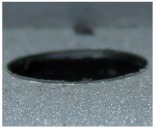	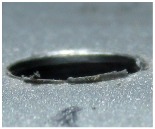
Twist drill with 2 edges	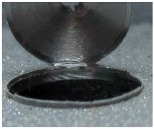	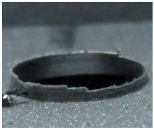	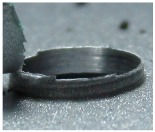
Twist drill with 3 edges	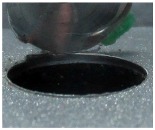	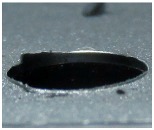	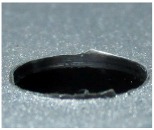
Pilot hole 2 mm and Twist drill with 2 edges	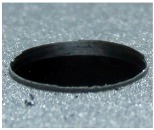	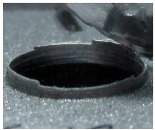	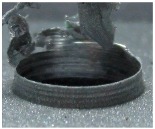
Pilot hole 3 mm and Twist drill with 2 edges	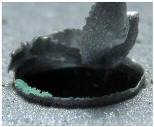	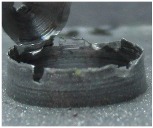	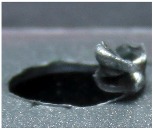
Pilot hole 4 mm and Twist drill with 2 edges